# Assessing pre-eclampsia awareness among pregnant women in Syria: a cross-sectional study on knowledge and perceptions

**DOI:** 10.1186/s12884-024-06368-4

**Published:** 2024-03-07

**Authors:** Haidara Bohsas, Hidar Alibrahim, Sarya Swed, Yasmeen Abouainain, Ahmed Aljabali, Samaa Masoud, Heba Haj Saleh, Tony Aldawoud, Fahima Taleb, Raneem Alnassif Alsheikh, Hassan Fawaz, Danya Mourad, Waleed Farouk Mohamed, Reham Aboushady, Wael Hafez

**Affiliations:** 1 Faculty of Medicine, Aleppo University, Aleppo, Syria; 2 Faculty of Medicine, University of Jordan, Amman, Jordan; 3 University of Science and Technology, Ar-Ramtha, Jordan; 4 Faculty of medicine, Damascus university, Damascus, Syria; 5 Al-hawash private university, Al-Mouzeina, Syria; 6 Healthpoint Hospital, Abu Dhabi, United Arab Emirates; 7 NMC Royal Hospital, 16th Street, Khalifa City, Abu Dhabi, UAE; 8 Medical Research Division, Department of Internal Medicine, The National Research Centre, Cairo, Egypt

**Keywords:** Pre-eclamsia, Awareness, Morbidity, Pregnancy, Pregnant women, Mortality, Socioeconomic, Syria

## Abstract

**Background:**

Pre-eclampsia (PE) is a major contributor to morbidity and mortality in mothers worldwide. Adequate understanding of this condition improves treatment, control, and prevention. This study evaluated preeclampsia awareness among pregnant women in Syria, and the characteristics related to awareness adequacy.

**Methods:**

This national cross-sectional study was conducted in Syria between 25 October and November 19, 2022. We included pregnant females of all age groups from all Syrian governorates. The questionnaire consisted of sociodemographic characteristics and knowledge of pre-eclampsia and its associated factors, symptoms, and complications.

**Results:**

Overall, 706 participants were involved in this research, with a mean age of 38.22. Only 52.1% of them reported that they had heard of preeclampsia. Among the participants, 56.5% stated that they would not terminate a pregnancy if they were determined to be likely to develop preeclampsia, while nearly 55.2% agreed to continue the pregnancy rather than deliver prematurely even if their where a potential risk on their health risks. Participants who reported a family history of PE or had already experienced PE were more likely to have appropriate preeclampsia knowledge than those who did not (OR = 2.27, OR = 3.18, respectively). Respondents aged 25 to 35 years had the highest knowledge scores, and participants living in cities scored higher knowledge than rural residents.

**Conclusion:**

According to our findings, pregnant women in Syria have a awareness gaps regarding the PE topic. This highlights the need to enhance women’s preeclampsia understanding for better pregnancy outcomes. Education through organizations, the media, and national programs is a significant aspect that promotes an adequate understanding of preeclampsia.

**Supplementary Information:**

The online version contains supplementary material available at 10.1186/s12884-024-06368-4.

## Introduction

Preeclampsia (PE) is characterized by high blood pressure and proteinuria at 300 mg/day in pregnant women [1]. This condition is one of the primary causes of maternal and child mortality in low-income countries and is accompanied by symptoms, such as edema, hypertension, and proteinuria [2]. As per the assessment conducted by the World Health Organization (WHO), the prevalence of preeclampsia spans from 2 to 10% across global pregnancies. Nevertheless, developing countries exhibit a reported incidence ranging from 1.8 to 16.7% [3].

Although the exact pathogenic mechanism of preeclampsia remains unclear, some animal and human studies have suggested that defective trophoblast invasion coupled with reduced uteroplacental perfusion may be the underlying cause [4].

Preeclampsia poses a variety of risk factors, including a history of preeclampsia, pregestational diabetes mellitus, chronic high blood pressure, antiphospholipid syndrome, and obesity. Furthermore, other critical factors contribute to the risk, such as advanced maternal age, nulliparity, history of chronic kidney disease, and use of assisted reproductive technologies [5]. Preeclampsia includes two stages: stage I is pre-clinical, which includes abnormal development of the uterine circulation, and stage II is the clinical stage, which manifests as the development of preeclampsia by relaying factors into the maternal circulation. The link between these two stages, proposed in 1993 by Roberts and Redman as endothelial dysfunction, suggests that the maternal endothelium serves as the primary target (leading to the development of stage II hypertension and proteinuria) for placenta-derived factors (generated in stage I) [6].

Preeclamptic pregnancies increase the long-term risk of cardiovascular disease and death in women, with the risk ranging from double the norm to an 8- to 9-fold increase in those who gave birth before 34 weeks of gestation [7]. The diagnosis of PE requires a new onset of hypertension and proteinuria during the second half of gestation. However, their presence does not always lead to complications and does not fully predict the serious consequences of PE. Current efforts focus on identifying this disorder, its pathogenesis, and its multisystemic nature to improve the prediction of early diagnosis and estimation of prognosis for maternal and fetal survival [8].

Adequate knowledge of preeclampsia is essential for the early detection, management, and prevention of adverse maternal and fetal outcomes. However, a significant knowledge gap remains concerning pre-eclampsia in low-income countries, preventing the implementation of effective preventive measures and timely interventions to improve maternal and neonatal health. Prior studies have asserted that informing patients about the disease has great benefits in complying with treatment and in reducing complications associated with the disease [9]. To address these problems, we need to assess women’s attitudes toward predictive and diagnostic tests and how they can influence decisions about treatment and early delivery [10].

Syria is middle east country that suffered from prolonged political and socioeconomic crisis for 11 years, leading to a marked decline in living standards and a substantial degradation of the healthcare infrastructure. The accessibility of healthcare services is notably constrained due to security-related impediments. Furthermore, the provision of maternal and child health services at the primary healthcare (PHC) level is disrupted. The ramifications for maternal and child morbidity and mortality stemming from deliveries occurring amidst the conflict are not definitively ascertained [11]. 

Given the importance of this matter on the lives of pregnant women and their fetuses and the absence of previous studies rating the awareness of pregnant Syrian women towards preeclampsia, we conducted this study to assess the knowledge and attitudes of pregnant Syrian women towards preeclampsia.

## Methods

### Study design and settings

This descriptive, survey-based, cross-sectional study was conducted in Syria between October 25 and November 19, 2022. Situated in the northern part of the Middle East in Southwest Asia, Syria is a relatively small country.

This study focused on Syrian women with at least one previous pregnancy across all age groups in every Syrian governorate, and we excluded non pregnant females, pregnant women working in the health sector, and non-Syrian females. Participation was voluntary, and those willing to contribute were required to indicate their informed consent by responding to the initial survey question, “Would you like to participate in this study?” To ensure the precision of the study, non-Syrian pregnant women, non-pregnant females, and individuals who declined participation were excluded from the analysis.

Before participating in the study, the participants received comprehensive information outlining the study’s objectives, significance, and procedural details. A clear emphasis was placed on the voluntary nature of their involvement, emphasizing their right to withdraw from the study at any point without repercussions. Confidentiality was maintained meticulously, ensuring all shared information would be strictly utilized for research purposes.

The sample size for this online survey was determined using a single-population proportion formula: n = [(Zα/2)² * P * (1—P)] / d².

- Confidence level = 95%.
- Z a/2 = 1.96.
- Margin of error = 5%.
- P = Proportion to be estimated = 50%.

The sample size is equal to (385). On the Google form website (https://www.google.com/forms/), (1046) participants were invited to complete the survey; however, (340) respondents declined, resulting in a final sample size of 706.

### Data Collection and Study Instrument

A web-based questionnaire was incorporated into a previous study [9]. The questionnaire was initially created in English using a back-translation technique, and then translated into Arabic. A professional healthcare translator has translated the English version into Arabic and then translated the Arabic version back to English for comparison with the original version. An Arabic online form was disseminated to potential participants via social media, including Facebook, Telegram, and WhatsApp, to ensure data security. Sixteen medical students from various medical schools in Syria performed the data collection (Data Collection Group). We applied a snowball sampling technique in the facilitation of data acquisition. A proficient investigator monitored the data collection process to uphold the integrity and dependability of the amassed data while concurrently insuring its confidentiality.

Two sections of the questionnaire were included. The first section measures sociodemographic characteristics, while the second evaluates knowledge of pre-eclampsia and its associated factors, symptoms, and complications.

A pilot study was conducted to confirm the validity and clarity of this questionnaire. The questionnaire was distributed to 47 expectant women, who were not part of the primary study. After designing the pilot study, we adopted a questionnaire and assured high internal consistency (Cronbach’s alpha varied from 0.712 to 0.861).

#### Sociodemographic variables

This section comprises 10 questions about participants’ sociodemographic variables, including age, residency, educational level, gestational age, whether this was their first pregnancy, the number of children they had, marital status, social status, whether they had experienced preeclampsia, and if there was a family history of preeclampsia.

#### Knowledge of preeclampsia and its associated factors, symptoms and complications

The participants’ knowledge of PE was evaluated through questions about awareness, signs/symptoms, risk factors, and complications. The questionnaire used 22 closed-ended questions with predefined response options (Additional File 1). For example, participants were asked, ‘What are some of the risk factors for preeclampsia?’ with answer choices like “Obesity [Yes], [No], and [I do not know].” Each correct response was awarded one point, whereas incorrect or unanswered questions received zero points. These scores were then converted to percentages, and the Bloom cutoff point was used to categorize preeclampsia knowledge into three levels: low (< 60%), medium (60–80%), and high (80–100%).

### Ethical consideration

Ethical clearance was obtained from the Syrian Ethical Society for Scientific Research in Aleppo (IRB number: FCL/P-17). The standard question was set at the beginning of the first page of the survey (Do you agree to participate in this study?). Participants who answered ‘Yes’ were automatically directed to the following sections, which offered detailed study questions. The estimated completion time of the survey was 10–14 minutes, and each answer was stored in a secure online database.

### Statistical analysis

Data were cleaned using Microsoft Excel before being exported to SPSS version 26 for analysis. Descriptive statistics, including simple frequencies and percentages, were used to analyze demographic characteristics and categorical variables, while continuous variables were represented as mean ± standard deviation. Logistic regression analysis was used to examine factors linked to sufficient preeclampsia knowledge. Statistical significance was set at *p* < 0.05.

## Results

### Sociodemographic characteristics and history of PE

This investigation incorporated a total of 706 subjects. The average age within the study populace was 38.22 ± 10.79 years, with a predominant residence in urban locales observed among more than two-thirds (*n* = 487, 69%) of the participants. Approximately (*n* = 429, 60.8%) of the participants possessed a university-level education, whereas (*n* = 44, 6.2%) exhibited a state of illiteracy. Most respondents (*n* = 378, 53.5%) conveyed a characterization of moderate economic circumstances. The prevalence of a familial history of preeclampsia was reported by a significant majority of study participants (*n* = 626, 88.7%). In comparison, only (*n* = 56, 7.9%) had previously received a diagnosis of preeclampsia (Table 1).

**Table 1:** Sociodemographic characteristics and history of PE

Variable	Number (n)	Percentage (%)
**Age (years), mean (± SD)**	38.22 (± 10.79)	
**Marital status**		
Married	658	93.2
Divorced	25	3.5
Widowed	23	3.3
**Employment status**		
No	393	55.7
Yes	313	44.3
**Residence status**		
Rural	219	31
Urban	487	69
**Education status**		
Illiterate	44	6.2
Primary	77	10.9
Secondary	156	22.1
University	429	60.8
**Income status**		
Bad	75	10.6
Moderate	378	53.5
Good	232	32.9
Excellent	21	3
**Experienced PE before?**		
No	650	92.1
Yes	56	7.9
**Family history of PE**		
No	626	88.7
Yes	80	11.3

### Knowledge of PE, risk factors, symptoms, and complications

A substantial number of participants reported being familiar with preeclampsia (*n* = 368, 52.1%). The most reported symptoms of preeclampsia were hypertension during pregnancy (*n* = 383, 54.2%), ongoing headache (*n* = 300, 42.5%), and feeling nauseous and wanting to vomit (*n* = 292, 41.4%). The predominant risk factor identified for preeclampsia was a prior history of the disorder (*n* = 320, 45.3%). Prevalent complications linked to preeclampsia encompassed both infant and maternal fatality (*n* = 405, 57.4%) and (*n* = 328, 46.5%), respectively. Furthermore, a significant proportion of the participants (*n* = 249, 35.3%) stated that they did not know the severity of the PE. (Table 2).

**Table 2:** Participants’ response to questions on knowledge of PE, risk factors, symptoms, and complications

Response	Number (n)	Percentage (%)
Are you familiar with the condition known as preeclampsia?		
No	338	47.9
Yes	368	52.1
**What are the signs and symptoms associated with preeclampsia?**		
Hypertension during pregnancy	383	54.2
Ongoing headache	300	42.5
Swelling	246	34.8
Visual impairment (blurry vision)	231	32.7
Pain in the chest	129	18.3
Pain in the abdomen	255	36.1
Feeling nauseous and want to vomit	292	41.4
Pain in the back	198	28
**What are the identified risk factors associated with the development of preeclampsia?**		
A history of preeclampsia in the family	292	41.4
A history of preeclampsia	320	45.3
Overweight and being obese	245	34.7
Diabetes mellitus	246	34.8
Adopting unhealthy life habits	282	39.9
Multiple infants born	190	26.9
**What complications are associated with preeclampsia?**		
Mother fatality	328	46.5
Infant fatality	405	57.4
Cardiac problems	161	22.8
dysfunction of the kidneys	194	27.5
**When is a woman most likely to develop preeclampsia?**		
Pregnancy that has reached a gestational age of 20 weeks or more.	379	53.7
**What is the severity of preeclampsia?**		
I don’t know	249	35.3
Not a major	21	3
Severe	200	28.3
Extremely severe	236	33.4
**Are you cautious regarding preeclampsia?**		
No	82	11.6
Yes	355	50.3
I don’t know	269	38.1

### Factors associated with adequate knowledge of PE among the study population

Four out of ten predictor variables were significantly correlated with good knowledge of preeclampsia (*P* < 0.05), including education, economic condition, family history of preeclampsia, and having prior preeclampsia. University-educated respondents showed a higher probability of having good knowledge of preeclampsia than did illiterate (AOR = 3.26; CI: 95%; P value < 0.05). Regarding financial status, participants with excellent income were more likely to have good knowledge than those with bad income (AOR = 3.29 CI: 95%; P value < 0.05). Respondents with a family history of preeclampsia and prior preeclampsia were more likely to have good preeclampsia knowledge than those who did not (AOR = 2.27 CI: 95%; P value < 0.05) (AOR = 3.18 CI: 95%; P value < 0.05) (Table 3).

**Table 3:** Factors associated with good knowledge of PE among the study population (good versus bad knowledge)

Variable		95% Confidence Interval		P value
	Adjusted Odds ratio (aOR)	Lower	Upper	
Age (years)				
25–35 – < 25	1.343	0.7055	2.558	0.369
> 35 – < 25	0.832	0.4233	1.635	0.594
**Marital status**				
Divorced – Married	1.039	0.4258	2.536	0.933
Widowed – Married	0.902	0.3358	2.425	0.839
**Employment status**				
Yes – No	1.067	0.7445	1.529	0.724
**Residence status**				
City – Countryside	1.193	0.8231	1.73	0.351
**Education status**				
Primary – Illiterate	0.789	0.319	1.951	0.607
Secondary – Illiterate	1.262	0.5559	2.866	0.578
University – Illiterate	3.261	1.4192	7.495	0.005
**Income status**				
Moderate – Bad	2.112	1.1041	4.039	0.024
Good – Bad	2.29	1.1491	4.563	0.019
Excellent – Bad	3.291	1.0492	10.323	0.041
**Parity**				
> 2–0–2	1.388	0.9486	2.03	0.091
**Family history of PE?**				
Yes – No	2.279	1.2888	4.029	0.005
**Experienced PE before?**				
Yes – No	3.189	1.5555	6.538	0.002
**Chronic diseases**				
Yes – No	1.486	0.9722	2.27	0.067

### Participants’ attitudes

Most of the participants (*n* = 566, 80.2%) expressed a willingness to undergo predictive testing, with over half (*n* = 399, 56.5%) indicating a commitment to continue conception even if the predictive test suggested a predisposition to preeclampsia. Regarding diagnostic assessments, a majority of respondents (*n* = 524, 74.2%) expressed consent to undergo such tests during pregnancy, citing the resultant peace of mind as a motivating factor, while 55.7% declined that diagnostic testing would increase their anxiety. Concerning the management of preeclampsia, a substantial percentage (*n* = 548, 77.6%) asserted a readiness to modify their dietary habits to mitigate the risk of preeclampsia despite the absence of conclusive empirical support for its efficacy. Similarly, a comparable proportion (*n* = 506, 71.7%) expressed a willingness to explore alternative treatments, even those entailing potential side effects to diminish the likelihood of encountering preeclampsia. Furthermore, regarding expectant management, (*n* = 397, 56.2%) of respondents advocated for immediate delivery in the case of severe preeclampsia diagnosis, while almost an equivalent proportion (*n* = 390, 55.2%) endorsed the continuation of pregnancy, even in the face of associated health risks, rather than opting for premature delivery (Table 4).

**Table 4:** Participants’ attitudes

Question	Responses		
		Frequency (n)	Percentage (%)
Participants’ views about prediction tests			
**1.** I would like to take an early test during my pregnancy that provides me with an estimate of my chances of developing a problem like preeclampsia, even if the prediction is not 100% accurate.	Agree	566	80.2
	Disagree	140	19.8
**2.** If a simple blood test had been available during my previous pregnancy(ies) that could predict whether I would develop preeclampsia, we may have made different decisions regarding the management of my pregnancy.	Agree	538	76.2
	Disagree	168	23.8
**3.** If there was a test in my first trimester that could predict with certainty that I would develop preeclampsia, I would consider ending my pregnancy.	Agree	307	43.5
	Disagree	399	56.5
**4.** If the prediction test indicated a low risk of developing preeclampsia, I would be more at ease with my prenatal care.	Agree	455	64.4
	Disagree	251	35.6
**5.** Having a test that predicts if I will develop preeclampsia in the future would provide me with some peace of mind during my pregnancy.	Agree	412	58.4
	Disagree	294	41.6
**6.** The prospect of undergoing a test to determine if I might develop preeclampsia at a later stage during pregnancy has caused me to feel anxious.	Agree	368	52.1
	Disagree	338	47.9
**7.** I would benefit from a test that could tell me whether I am likely to develop preeclampsia in the latter stages of my pregnancy, even if there is presently no treatment for it.	Agree	407	57.6
	Disagree	299	42.4
**8.** A reliable prediction test that indicates whether I will develop preeclampsia is of such significance that I would be willing to pay a reasonable sum out of pocket, even if it was not part of my healthcare coverage.	Agree	480	68
	Disagree	226	32
**Participants’ views about diagnostic tests**			
**9.** The existing diagnostic approaches for preeclampsia are deemed satisfactory.	Agree	419	59.3
	Disagree	287	40.7
**10.** A diagnostic test for preeclampsia during pregnancy would provide me with a sense of tranquility.	Agree	524	74.2
	Disagree	182	25.8
**11.** A diagnostic test for preeclampsia during pregnancy would increase my anxiety	Agree	313	44.3
	Disagree	393	55.7
**12.** A diagnostic test for preeclampsia during pregnancy would be valuable to me, despite the fact that there is currently no cure for the condition.	Agree	472	66.9
	Disagree	234	33.1
**13.** A diagnostic test that can accurately determine whether I have preeclampsia is of such importance to me that I would be willing to pay a reasonable amount out of pocket, even if it was not covered by my healthcare plan.	Agree	482	68.3
	Disagree	224	31.7
**Participants’ views about treatment for HDPs**			
**14.** I would be apprehensive about taking a baby aspirin every day to minimize my risk of developing preeclampsia without having confidence that it would not have any adverse effects on my baby in the long run.	Agree	437	61.9
	Disagree	269	38.1
**15.** While there is no scientific evidence to establish a correlation between a woman’s diet and the likelihood of developing preeclampsia, I am willing to make substantial changes to my diet in an effort to minimize my risk.	Agree	548	77.6
	Disagree	158	22.4
**16.** I am willing to explore other treatment options, even if they have potential side effects, to lower my risk of developing preeclampsia.	Agree	506	71.7
	Disagree	200	28.3
**17.** Given that baby aspirin has been demonstrated in certain studies to be safe and effective in reducing the risk of preeclampsia in some women, I am willing to take it throughout my pregnancy, even if it may not provide any benefit to me personally.	Agree	467	66.1
	Disagree	239	33.9
**18.** If it could prevent preeclampsia, I am willing to participate in a clinical trial testing a medication that has been shown to be safe for the infant but has never been administered to expectant women.	Agree	365	51.7
	Disagree	341	48.3
**19.** The possibility of developing preeclampsia is a greater concern of mine than the potential risks associated with preventative treatments, in terms of potential impact on my health.	Agree	383	54.2
	Disagree	323	45.8
**20.** In considering potential treatments to enhance my pregnancy outcomes, I am more concerned with ensuring the safety of the treatment for my unborn baby’s wellbeing than for my own.	Agree	485	68.7
	Disagree	221	31.3
**Participants’ views about expectant management**			
**21.** If I am diagnosed with severe preeclampsia, I am uncomfortable with doctors trying to determine the most appropriate time to deliver the baby in order to ‘buy time’, as I believe it is best to deliver the baby as soon as possible.	Agree	397	56.2
	Disagree	309	43.8
**22.** Should I ever be presented with a decision to make, I would choose to carry my pregnancy to term, even if doing so put my own health at risk, rather than bringing my baby into the world prematurely.	Agree	390	55.2
	Disagree	316	44.8

### Correlation Matrix

A statistically significant moderate positive association was found between age and parity (*r* = 0.53, *p* < 0.001), whereas there was a significant weak negative correlation between the total knowledge score and parity (*r*=-0.10, *p* = 0.007) (Table 5).

**Table 5:** Correlation Matrix

Variables	Correlational coefficients	Age	TKS	Parity
Age	Spearman’s rho	—		
	p-value	—		
	N	—		
TKS	Spearman’s rho	-0.066	—	
	p-value	0.08	—	
	N	706	—	
Parity	Spearman’s rho	0.534***	-0.102**	—
	p-value	< 0.001	0.007	—
	N	706	706	—

*Note* * *p* < 0.05, ** *p* < 0.01, *** *p* < 0.001

### Differences in total knowledge score (TKS) among different sociodemographic characteristics

There were significant associations between the five variables of different sociodemographic characteristics (age group, employment status, residence status, education status, and income status) and the total knowledge score of preeclampsia (*P* < 0.05). Respondents aged 25 to 35 had the highest total knowledge score compared to the other group (3.58 ± 2.24). Additionally, respondents living in cities scored higher on knowledge than rural residents (3.48 ± 2.26). Regarding the education status, illiterate respondents were less knowledgeable than educated participants in all educational subgroups(2.16 ± 2.27). However, participants with bad income status had the lowest total knowledge score among other economic groups (2.24 ± 2.3). On the other hand, the study participants with prior preeclampsia and a family history of PE had higher total knowledge scores than those who did not (4.75 ± 1.82) and (4.51 ± 1.9), respectively(Table 6).

**Table 6:** Differences in total knowledge score (TKS) among different sociodemographic characteristics

Variable	Mean Knowledge score (SD), overall = 3.32 (2.26)	P value
Age groups (years)		0.043
< 25	3.35 (2.266)	
25–35	3.58 (2.242)	
> 35	3.12 (2.257)	
**Marital status**		0.210
Married	3.36 (2.26)	
Divorced	2.8 (2.16)	
Widowed	2.74 (2.281)	
**Employment status**		< 0.001
No	2.92 (2.221)	
Yes	3.81 (2.213)	
**Residence status**		0.004
Countryside	2.95 (2.215)	
City	3.48 (2.262)	
**Education status**		< 0.001
Illiterate	2.16 (2.272)	
Primary	2.19 (2.14)	
Secondary	2.63 (2.194)	
University	3.88 (2.116)	
**Income status**		< 0.001
Bad	2.24 (2.307)	
Moderate	3.27 (2.198)	
Good	3.68 (2.23)	
Excellent	4.05 (2.291)	
**Experienced PE before?**		< 0.001
No	3.19 (2.252)	
Yes	4.75 (1.822)	
**Family history of PE**		< 0.001
No	3.16 (2.257)	
Yes	4.51 (1.903)	

## Discussion

Preeclampsia (PE) is a prevalent contributor to maternal morbidity and mortality on a global scale, impacting an estimated 2–8% of pregnancies worldwide, with rates escalating to 10% in low-income nations [12]. Given the profound implications of this condition for both pregnant women and their fetuses, coupled with the dearth of prior investigations assessing the awareness of preeclampsia among pregnant Syrian women, this study was undertaken to assess their comprehension of this medical concern. The gravity of preeclampsia in jeopardizing the well-being of pregnant women and their unborn offspring motivated our inquiry. Our findings revealed that 52.1% of all participants demonstrated an awareness of preeclampsia. In contrast, a study conducted in Ghana [9] reported that 88.4% of respondents exhibited inadequate awareness of preeclampsia. Nonetheless, a minority of respondents in both our study and the Ghanaian research possessed limited insights into the signs, symptoms, risk factors, and consequences of preeclampsia. This observation underscores the need for further investigation to elucidate the potential underlying factors that could inform the development of pertinent health promotion interventions. Based on sociodemographic characteristics, age between 25 and 35 years, city residency, university-level education, good income, prior preeclampsia, and family history of preeclampsia were significantly associated with greater odds of having adequate knowledge of preeclampsia. Participants who had experienced prior preeclampsia and those with a family history of preeclampsia were significantly more likely to have adequate knowledge about preeclampsia. Similarly, having a higher level of education and being of a more advanced age were substantially linked with better chances of having a sufficient understanding of PE [13]. Because patients’ knowledge of an illness favorably affects their compliance to treatment and helps abate difficulties connected with the condition, evidence suggests that a good understanding of a disorder helps with its prevention, control, and management [13, 14]. For instance, MacGillivray et al. conducted an intervention trial in Jamaica and found that providing cards depicting the symptoms of PE resulted in fewer adverse events occurring among patients [15]. This demonstrates that having a solid understanding of preeclampsia is directly related to better clinical results, and that the opposite is true. Therefore, determining the prevalence of preeclampsia among high-risk populations and improving their understanding of the condition may be vital for reducing the rising incidence of the illness and its associated adverse effects.

When women are informed about the potential outcomes of the symptoms they experience, there is a greater likelihood that they will seek urgent medical attention. The low levels of awareness of preeclampsia found in this study are concerning; nevertheless, they are not insurmountable because the variables that impacted those levels of knowledge were not fixed or general demographic characteristics. Having a high educational level, being economically well off, having a family history of preeclampsia, and having previously experienced preeclampsia were the only factors independently associated with adequate knowledge of preeclampsia after adjusting for other factors that could influence the relationship. This suggests that educating women about preeclampsia through effective methods, such as during antenatal visits, media channels, and with family members, could help improve patients’ understanding of the condition and potentially reduce preeclampsia-related deaths in Syria, similar to the results of a study conducted in Ghana [9], where a higher educational level was found to be associated with better knowledge of preeclampsia. Enhancing patient awareness of preeclampsia has been demonstrated to result in earlier reporting of symptoms, potentially leading to more rapid intervention and better health outcomes for both mother and baby [16, 17]. You et al. proposed that a significant reduction in the severe consequences of preeclampsia might be achieved by increasing the knowledge of the condition and reporting symptoms at an earlier stage [18]. Similarly, research conducted in the United States by Ogunyemi et al. suggested that patient education could have prevented 72% of the instances of eclampsia [19]. Furthermore, due to the plausible relationship between preeclampsia knowledge and improved clinical outcomes, these earlier findings support our argument that improving knowledge of preeclampsia among pregnant women is crucial for reducing the prevalence, complications, and mortality associated with the disease.

Our findings underscore the need to enhance the awareness and knowledge of preeclampsia among pregnant Syrian women. Despite its significant impact on maternal and fetal health, a substantial knowledge gap exists regarding preeclampsia symptoms, risk factors, and complications. Tailored educational initiatives are crucial to address this gap, especially for those with lower education levels and limited healthcare access. These programs should be integrated into antenatal care, community workshops, and digital platforms, offering comprehensive information to empower women to make informed decisions regarding their health.

Moreover, we should highlight the importance of mitigating the anxiety associated with diagnostic tests by fostering effective communication and psychological support. Shared decision making should be promoted among healthcare professionals and pregnant women to ensure that treatment choices align with individual preferences and evidence-based guidance. In the long term, policy efforts must improve the healthcare infrastructure and equitable access to care, guaranteeing that all pregnant women have equal opportunities for education, early detection, and proper management of preeclampsia. By addressing these recommendations, healthcare systems can make significant strides toward reducing the burden of preeclampsia on maternal and neonatal health in Syria and similar contexts.

The study’s inability to fully explore participants’ answer options in light of their pre-existing knowledge was constrained by closed-ended questions. Women selected to participate in the research after making an in-person visit to the clinic or hospital may have different characteristics than women who had consultations by phone, creating the possibility of selection bias. The second set of females may have responded differently, leading to different research results. There is also the possibility of skewed results because the survey was administered online to those who were more likely to be college-educated, have their own cell phones, and live in urban areas. The cross-sectional study design presents potential bias and cannot prove a causal relationship. The utilized sample size surpassed the prescribed quantity for the investigation to guarantee adequacy, addressing the potential occurrence of recurrent or unfinished responses. The economic status has not categorized according to a certain. This online cross-sectional study employed social media platforms as a means of data acquisition; although a proficient investigator oversaw the data collection, the potential for bias remains inherent. The sample, drawn from various Syrian governorates, is limited in size, thereby precluding its comprehensive representation of the entirety of the Syrian female population.

## Conclusion

Our investigation identified knowledge lacunae pertaining to preeclampsia among Syrian women. Nevertheless, education plays a crucial role in augmenting awareness and understanding of this ailment. Notably, personal or familial history of preeclampsia emerges as the most pivotal determinant for fostering a comprehensive comprehension of the condition. This underscores the imperative to intensify attempt aimed at enlightening women about preeclampsia to enhance the prospects of healthier pregnancies. The dissemination of information can be derived from diverse channels such as contextual health education administered during antenatal care, media platforms, and national educational initiatives. The amplification of educational campaigns is recommended to bolster awareness on the subject. Furthermore, healthcare providers specializing in obstetrics are well-positioned to contribute to education by imparting knowledge to pregnant individuals attending their clinics or hospitals, thereby mitigating the risk of severe complications associated with this disease.

### Electronic supplementary material

Below is the link to the electronic supplementary material.

**Supplementary Material 1:** The questionnaire tool

## Data Availability

The authors have access to and saved all the data necessary to support the conclusion of this paper. All data are accessible upon reasonable request from the corresponding author.
